# Icariin ameliorates minimal change disease by regulating the mitochondrial dysfunction pathway: an integrated strategy of network pharmacology, bioinformatics, and experimental validation

**DOI:** 10.3389/fphar.2025.1640822

**Published:** 2025-08-29

**Authors:** Hao Wu, Rong Wu, Dian Zhong, Enlai Dai, Li Chen, Guozhong Xue, Xuping Li, Hanyu Wang

**Affiliations:** ^1^ School of Traditional Chinese and Western Medicine, Gansu University of Chinese Medicine, Lanzhou, Gansu, China; ^2^ Department of Nephropathy, Affiliated Hospital of Gansu University of Chinese Medicine, Lanzhou, Gansu, China; ^3^ Department of Nephropathy, The First Hospital of Lanzhou University, Lanzhou, Gansu, China; ^4^ Pharmaceutical Preparation Section, The Second People’s Hospital of Baiyin Municipality, Baiyin, Gansu, China

**Keywords:** icariin, mitochondrial dysfunction, minimal change disease, key genes, network pharmacology

## Abstract

**Background:**

Minimal change disease (MCD) involves mitochondrial dysfunction. Icariin (ICA) has therapeutic potential. However, the exact mechanism by which ICA regulates mitochondrial dysfunction remains to be elucidated. This study investigated ICA targets and mitochondrial dysfunction-related genes (MDRGs) involved in MCD pathogenesis.

**Methods:**

First, the differentially expressed genes (DEGs) between MCD and controls were identified using differential expression analysis. Differential MCD-ICA target genes were obtained by intersecting the DEGs and MDRGs with ICA target genes. The four Cytoscape algorithms were then used to screen the differential MCD-ICA target genes for candidates, which were then refined through expression validation, machine learning, and ROC analysis to pinpoint the key genes. Next, a nomogram model of MCD was constructed. Gene set enrichment analysis (GSEA), immune infiltration analysis, molecular regulatory network analysis, and molecular docking analysis were also performed using the key genes. Finally, reverse transcription quantitative polymerase chain reaction (RT-qPCR) was used to validate the expression of the key genes in rat samples. In parallel, mitochondrial morphology was examined using transmission electron microscopy, and the ATP content in renal tissue was measured using colorimetric detection.

**Results:**

Two key genes (*ANPEP* and *XDH*) were identified; both were downregulated in MCD. These findings were confirmed using RT-qPCR, with ICA intervention reversing their expression. In addition, the key gene-based nomogram demonstrated good predictive ability. Molecular docking confirmed strong binding between ICA and each of the key genes. GSEA revealed that the top three most prominent pathways shared by the two key genes included neutrophil degranulation and the innate immune system, with differential immune cell infiltration noted between the MCD patients and controls (e.g., resting dendritic cells and eosinophils). Twelve transcription factors co-regulated the genes *XDH* and *ANPEP*. Transmission electron microscopy and colorimetry confirmed that the ICA intervention alleviated mitochondrial dysfunction.

**Conclusion:**

*ANPEP* and *XDH* were identified as associated with ICA therapy and MDRGs in MCD patients. Furthermore, the potential ameliorating effect of ICA on MCD could be achieved by alleviating mitochondrial dysfunction. This work provides a potential theoretical basis for the treatment of MCD.

## Highlights

1. This study identified two key genes (*ANPEP* and *XDH*) related to ICA and mitochondrial dysfunction in MCD. Molecular docking confirmed their high-affinity binding with ICA. RT-qPCR validated their dysregulation in MCD and ICA’s regulatory effects.
2. *ANPEP* was shown to modulate core enzymes in the glutathione (GSH) metabolic axis, thereby mediating oxidative stress cascades, while *XDH* was found to participate in NLRP3 inflammasome remodeling and maintain redox homeostasis.
3. Olfactory receptors may serve as novel mechano-transduction elements that participate in the dynamic regulation of the glomerular filtration barrier via certain mechanisms.
4. Twelve transcription factors were identified as co-regulating ANPEP and XDH, with KLF5 being particularly prominent in demonstrating their pathological crosstalk between renal fibrotic progression and inflammatory cascades.

## 1 Introduction

Minimal change disease (MCD), also called “lipoid nephropathy,” is an important pathological type of nephrotic syndrome (NS). Clinical manifestations of MCD include hypoproteinemia, massive proteinuria, peripheral edema, and hyperlipidemia (Kristensen et al., 2021). Massive proteinuria is caused by a breakdown of the glomerular filtration barrier. MCD accounts for approximately 10%–15% of all cases of primary nephrotic syndrome in adults and 70%–90% of all cases in children, with an increasing trend annually (Vivarelli et al., 2017). The underlying mechanisms contributing to MCD pathogenesis have not been elucidated to date. Factors such as immune dysfunction, mitochondrial damage, and genetic susceptibility may play central roles in MCD pathogenesis (Vincenti et al., 2023). Glucocorticoids are the first-choice drug for MCD treatment, which exhibit a remission rate of over 80%; however, relapse is common after remission (Bensimhon et al., 2019). Therefore, immunosuppressants, including mycophenolate mofetil (MMF), calcineurin inhibitors (CNI), and cyclophosphamide (CTX), are often used in combination. However, the long-term use of immunosuppressants has side effects (Gomez et al., 2024). In recent years, biological agents such as rituximab (RTX) and telitacicept have achieved certain therapeutic effects in the treatment of MCD (Lan et al., 2024; Li et al., 2023). However, the number of clinical studies on these biological agents is limited, and their long-term efficacy and safety must be explored and confirmed. Therefore, it is important to identify the potential novel markers to reduce the recurrence rate of MCD patients and improve the therapeutic effect in MCD treatment.

Icariin (ICA) is one of the main active components of ICA flavonoids, which are extracted from the Chinese herb ICA. ICA has many pharmacological effects, such as inhibiting osteoclasts, protecting the cardiovascular system, and enhancing immune function (He et al., 2020). Studies have shown that ICA can downregulate NOD3 and caspase-1 levels in rats and inhibit TGF-β and α-SMA levels in HK-2 cells, thereby alleviating renal interstitial fibrosis in NS (Duan et al., 2024). In the HK-2 cell model, ICA partially activates the Nrf2/HO-1 pathway, inhibits inflammatory factors, maintains mitochondrial morphology, reduces the excessive production of reactive oxygen species (ROS) and mtROS, and protects renal function (Ding et al., 2024). Furthermore, ICA has been demonstrated to alleviate renal fibrosis in chronic kidney disease by inhibiting IL-1β/TGF-β-mediated activation of renal fibroblasts (Wang et al., 2021). In summary, ICA exerts protective effects in multiple systems, including the nervous system, various tumors, and the kidneys, by modulating multiple signaling pathways, demonstrating promising therapeutic potential.

Mitochondria are essential for maintaining cellular homeostasis and serve as energy sources for cells. They also exert a key influence on kidney function. Mitochondrial dysfunction is considered a cause of glomerular and tubular diseases (Casa et al., 2014). Additionally, oxidative damage to proteins, particularly albumin, has been linked to immune pathogenesis in chronic diseases such as rheumatoid arthritis, suggesting that similar mechanisms may contribute to renal pathologies (Khan et al., 2018). Network pharmacology boosts the efficacy of clinical drug trials and reduces development costs by facilitating precise multitarget molecular design. Multiple pathway interventions in signaling cascades are employed to optimize therapeutic benefits and mitigate toxicity. Network pharmacology has advanced drug discovery by modeling system-level interactions between drugs and human biological networks and has been widely used to study the potential molecular underpinnings of the therapeutic and mechanistic effects of drug compounds and their bioactive constituents across multiple disease models (Aihaiti et al., 2021). In this study, based on the transcriptome data of MCD and the use of bioinformatics and network pharmacology, the key genes related to ICA therapy and mitochondrial dysfunction in MCD were investigated, and the potential molecular mechanisms of the key genes in MCD were explored, providing novel references for the diagnosis and follow-up treatment of MCD patients.

## 2 Methods

### 2.1 Data collection

Datasets of gene expression studies focusing on MCD were selected from the Gene Expression Omnibus (GEO) database (http://www.ncbi.nlm.nih.gov/geo/). These datasets were required to explicitly include renal tissue samples from MCD patients and healthy controls with complete and downloadable data, and their sample sizes had to meet the requirements for statistical analysis (training set sample size ≥20; validation set sample size ≥15). Ultimately, three datasets were selected: GSE216841, GSE246204, and GSE139061. The training set GSE216841 contained 35 samples. After excluding 13 samples of idiopathic membranous nephropathy, renal tissue samples from 14 patients with MCD and 8 healthy controls were selected for analysis. Kidney sample data of 12 MCD patients were obtained from GSE246204 (sequencing platform: GPL20301), and the data of 9 healthy control kidneys were obtained from GSE139061 (sequencing platform: GPL20301). The GSE139061 and GSE246204 data were merged into the validation dataset. A total of 3,278 mitochondrial dysfunction-related genes (MDRGs) were retrieved from the GeneCards database (https://www.genecards.org/) (relevance score >5) (Niu et al., 2024) (Supplementary Table S1). The structures of the icariin (ICA) compounds were obtained by searching in the PubChem database (https://pubchem.ncbi.nlm.nih.gov/) using the keyword “ICA,” and the chemical structures of the ICA compounds were uploaded to PharmMapper (http://lilab-ecust.cn/pharmmapper/), TargetNet (http://targetnet.scbdd.com/), the Comparative Toxicogenomics Database (CTD) (https://ctdbase.org//), the Encyclopedia of Traditional Chinese Medicine (ECTM) (http://www.tcmip.cn/ETCM2/front/#/), and the Traditional Chinese Medicines Systems Pharmacology Platform (TCMSP) (https://old.tcmsp-e.com/tcmsp.php) databases to predict ICA targets, remove duplicate targets, and convert target proteins to the genes based on UniProt IDs in the UniProtKB database (https://www.UniProt.org/). The toxicological parameters of ICA were determined using the ProTox II website (https://tox-new.charite.de/protox_II/).

### 2.2 Differential expression analysis

Differentially expressed genes (DEGs) between the MCD and control samples were screened from GSE216841 using the DESeq2 package (v 1.42.0) (Love et al., 2014). Gene names were standardized (probe IDs were converted to official gene symbols via the UniProt database, and duplicate or unannotated probes were removed), and low-expression genes were filtered out (only genes with expression levels >0 in ≥75% of samples were retained) to reduce background noise. The thresholds used were p < 0.05 and |log_2_-fold change (FC)| > 0.5. The ggplot2 package (v 3.3.2) (Gustavsson et al., 2022) was used to plot the volcano plot of these DEGs and mark the top ten upregulated and downregulated DEGs. The pheatmap package (version 0.7.7) (Gu et al., 2016) was used to plot a heatmap.

### 2.3 Identification and enrichment analysis of differential MCD-ICA target genes

The VennDiagram package (version 1.7.3) (Mao et al., 2022) was used to screen the differential MCD-ICA target genes by intersecting the DEGs, MDRGs, and ICA target genes. The clusterProfiler package (version 3.16.0) (Wu et al., 2021) was used for Gene Ontology (GO) and Kyoto Encyclopedia of Genes and Genomes (KEGG) enrichment analyses of differential MCD-ICA target genes (p < 0.05).

### 2.4 Protein–protein interaction (PPI) network

The interplay of the differential MDRGs we determined was explored, and the differential MDRGs were input into the STRING website for analysis. Cytoscape software (version 3.10.2) (Shannon et al., 2003) was used to visualize the PPI network (https://string-db.org/) (confidence = 0.4). The four algorithms (MCC, stress, MNC, and degree) in the cytoHubba plugin in Cytoscape software were used to screen for candidate genes with the top 25 scores for each algorithm. The genes screened using these four algorithms were then intersected using the VennDiagram package.

### 2.5 Machine learning

Random forest (RF), neural network (NNet), extreme gradient boosting (XGBoost), and support vector machine (SVM) models were built using the caret package (version 6.0–91) (Zhang et al., 2019). The residuals of the RF, NNet, XGBoost, and SVM models were analyzed using the DALEX package (version 2.4.3) (Guan et al., 2023). The classification efficacy of the four models was evaluated based on the area under the curve (AUC) scores in the training and validation sets using the pROC package (version 1.18.0) (Robin et al., 2011). The model with the lowest residuals and the highest AUC was selected as the best prediction model, and the top ten genes ranked using this model were selected as the characterized genes (AUC >0.7).

### 2.6 Identification of key genes

In the training (GSE216841) and the validation (GSE139061 and GSE246204) sets, the genes with consistent expression trends and significant differences (p < 0.05) between the groups were obtained using the Wilcoxon test for subsequent analysis. The ROC curves of each characterized gene in the training and validation sets were then visualized using the pROC package (version 1.18.0) (Robin et al., 2011), and the characterized genes with AUC values greater than 0.7 were defined as key genes. Correlation analysis of the key genes was performed using the rcorr function in the Hmisc package (version 5.1–3) (http://biostat.mc.vanderbilt.edu/s/Hmisc) (|correlation (cor)| > 0.3, p < 0.05).

### 2.7 Nomogram construction and evaluation

A nomogram of the key genes was constructed using the rms package (version 5.1–4) (Xu et al., 2023), and decision curves were visualized using the rmda package (version 1.6) (https://github.com/mdbrown/rmda). Calibration curves were visualized using the regplot package (version 1.1) (Zhang et al., 2018). The objective was to assess the fidelity of the nomogram.

### 2.8 Gene set enrichment analysis (GSEA)

To better understand the biological functions and pathways of the key genes involved in the process of MCD development, “c2.cp.kegg.v2023.1.Hs.symbols.gmt” was obtained from the Molecular Signatures Database (MSigDB) to serve as a background gene set. In GSE216841, the correlation coefficients (p < 0.05) between the key genes and other genes were calculated and ranked using the corrplot package (v 0.92) (Zhang et al., 2023). GSEA was subsequently conducted using the clusterProfiler package (version 3.16.0) (Wang et al., 2022) (adj.p < 0.05, |normalized enrichment score (NES)| > 1). Using the top five KEGG pathways of the key genes, an ICA–key gene–pathway network diagram was constructed using Cytoscape software (version 3.10.2) (Shannon et al., 2003).

### 2.9 Immune infiltration analysis

The proportions of 22 infiltrating immune cells between the MCD and control groups were analyzed using the CIBERSORT algorithm. The Wilcoxon signed-rank test was used to compare the differences (p < 0.05) in immune-infiltrating cells between the MCD and control groups. Differential immune cells and correlations between key genes and immune cells were determined based on the results of Spearman analysis using the psych package (version 2.2.5) (Robles-Jimenez et al., 2021).

### 2.10 Association analysis of key genes with mitochondrial dysfunction

Mitochondrial dynamics genes from the MitoMiner, MitoCarta, and NCBI GEO databases were collected, and the correlations between the 23 mitochondrial kinetic genes and the differential immune cells were determined based on Spearman correlation analysis results conducted using the psych package (version 2.2.5) (Robles-Jimenez et al., 2021).

### 2.11 Expression analysis of key genes in kidney tissue cells

The Human Protein Atlas (HPA) database (https://www.proteinatlas.org/) was used to analyze the expression of key genes in nine kinds of kidney tissue cells (podocytes, proximal tubular cells, ascending loops of Henle cells, intercalated cells, endothelial cells, fibroblasts, macrophages, T cells, and plasma cells). These were analyzed using the psych package (version 2.2.5) (Robles-Jimenez et al., 2021).

### 2.12 Construction of molecular regulatory networks

ChIP-X Enrichment Analysis 3 (ChEA3) (https://maayanlab.cloud/chea3/) was used to predict the TFs that targeted the determined key genes. The network depicting the TF–mRNA interactions was established using Cytoscape software (version 3.10.2) (Shannon et al., 2003).

### 2.13 Molecular docking

Molecular docking of drugs to the key genes was performed using CB-Dock, and visualization was performed using PyMOL software. The PDB database (https://www.rcsb.org/search/advanced/structure) was searched, and the protein structures of the key genes were downloaded. The molecular structure of ICA was obtained from the PubChem database (https://pubchem.ncbi.nlm.nih.gov/), and the binding energy was <–5 kcal/mol.

### 2.14 Reverse transcription quantitative polymerase chain reaction (RT-qPCR)

Nine frozen tissue samples from rats were collected from the Gansu University of Chinese Medicine, of which three samples were from rats with MCD, three were from control rats, and three were from MCD rats treated with ICA. The ethical approval authority was the Lanzhou Veterinary Research Institute, Chinese Academy of Agricultural Sciences (ethical approval number LVRIAEC-2024–074). Total RNA was extracted from these nine rat tissue samples using TRIzol reagent (Ambion, United States). The primer sequences are detailed in Table 1.

**TABLE 1:** Sequences of the primers used.

Primer	Sequence
*ALB* F	TTT​CCT​GTC​AAC​CCC​ACT​AGC
*ALB* R	TGG​GCG​ATC​TCA​CTC​TTG​TG
*ANPEP* F	CCC​TGG​TAA​AGG​GCC​ATC​AG
*ANPEP* R	AGG​ATT​TTC​GAG​CAT​CGG​CA
*XDH* F	ACT​GTA​GTG​GCT​CTC​GAG​GT
*XDH* R	CTC​CCA​GTG​CCT​CGA​ATG​TT
*GAPDH* F	GGC​CGG​AGA​CGA​ATG​GAA​ATT​A
*GAPDH* R	CCA​AAT​CCG​TTC​ACA​CCG​AC

RT-qPCR analysis was conducted on a CFXLFZ006 real-time PCR detection system (Bio-Rad, Shanghai, United States). The collected data were analyzed using the well-established 2^−ΔΔCT^ method, with GAPDH used as the reference gene for normalization (Livak and Schmittgen, 2001). Finally, GraphPad Prism (version 5.0.0) (Al-Rawi et al., 2023) was used to plot and calculate the p-value.

### 2.15 Colorimetric detection of ATP content

Kidney tissue samples from SD rats were homogenized, and mitochondria were isolated using differential centrifugation. The homogenate was lysed by adding 100–200 μL of lysis buffer per 20 mg of tissue, followed by conducting thorough homogenization using a glass homogenizer or an equivalent device. Complete tissue lysis was ensured through adequate homogenization. After lysis, the samples were centrifuged at 12,000 × g for 5 min at 4°C, and the supernatant was collected. An ATP assay kit (Beyotime, Cat. #S0026, Shanghai, China) was used according to the manufacturer’s instructions, and the reaction mixture was incubated in the dark at room temperature for 5 min. Absorbance at 560 nm was then measured using a microplate reader. The ATP concentrations in the samples were calculated based on an ATP standard curve.

### 2.16 Transmission electron microscopy analysis of renal ultrastructure

Kidney tissues were fixed in 4% glutaraldehyde for 4 h, rinsed with phosphate buffer, and post-fixed in 1% osmium tetroxide for 2 h. The samples were then dehydrated through a graded ethanol series, infiltrated with an acetone–epoxy resin mixture, and embedded in pure epoxy resin. Ultrathin sections were obtained from the samples and were then double-stained with uranyl acetate and lead citrate for electron microscopy observation.

### 2.17 Statistical analysis

**FIGURE 1:**
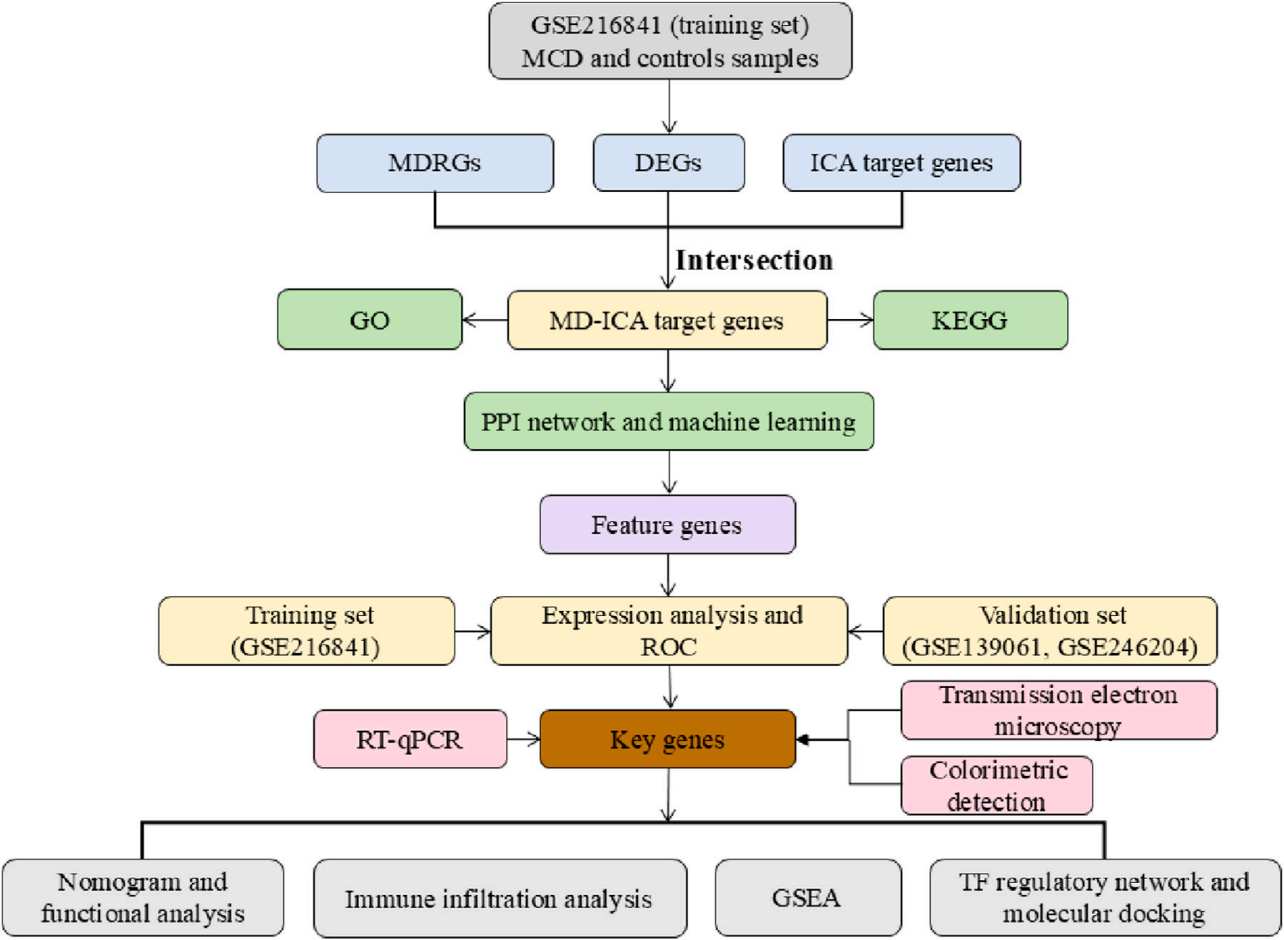
Analysis flowchart.

Bioinformatics analyses were performed using the R programming language (version 4.2.2). Differences between two groups were determined using the Wilcoxon rank-sum test, and differences between the PCR experimental groups were determined using the Mann–Whitney U test (p < 0.05). The analysis process of this study is detailed in Figure 1.

## 3 Results

### 3.1 Identification and functional analysis of differential MCD-ICA target genes

**FIGURE 2:**
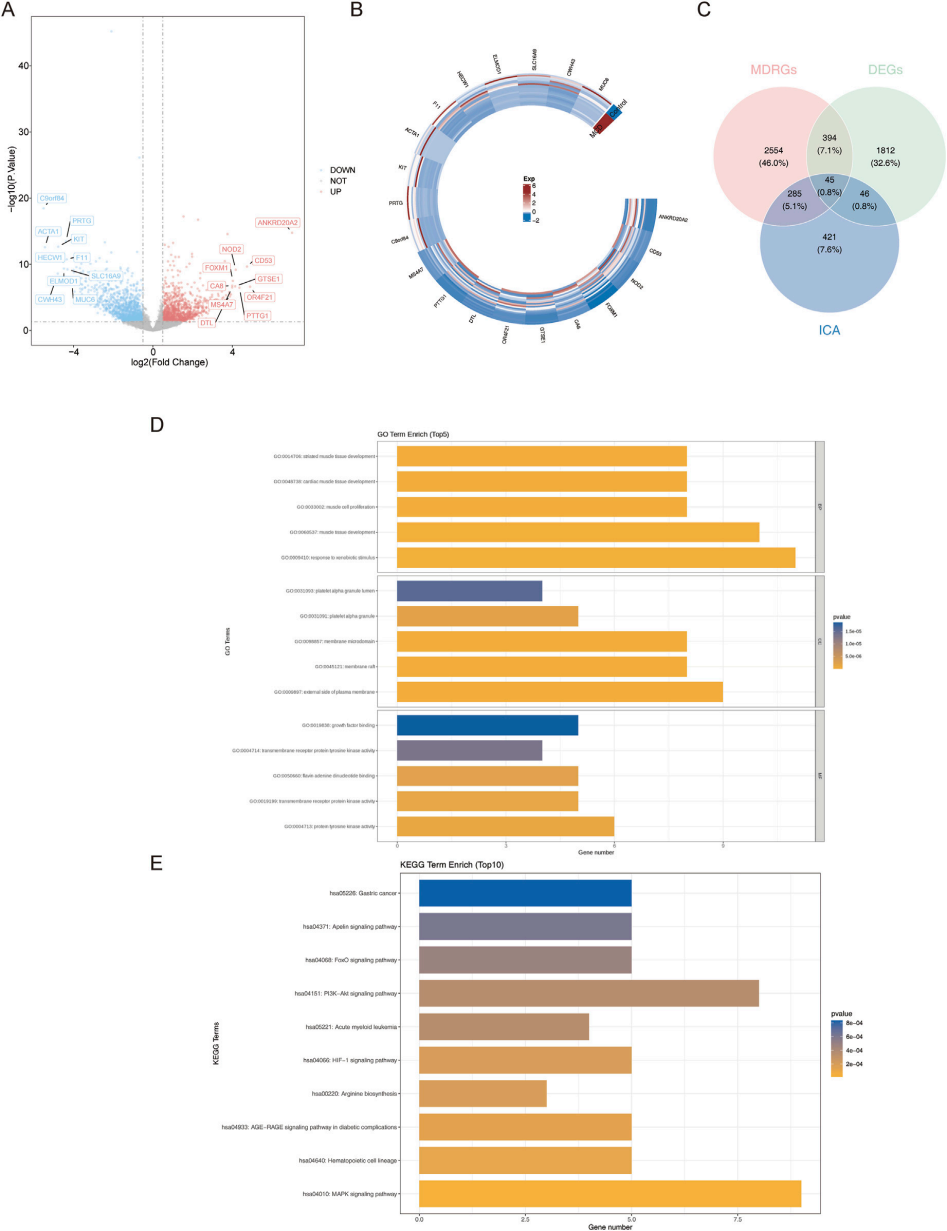
Identification and functional analysis of differential MCD-ICA target genes. **(A,B)** Volcanic map and heatmap of DEGs. **(C)** Screening of differential MCD-ICA target genes by intersecting DEGs, MDRGs, and ICA target genes. **(D,E)** GO and KEGG enrichment result plots of differential MCD-ICA target genes.

Data from GSE216841 were analyzed to identify the genes that were differentially expressed between minimal change disease (MCD) and normal samples. A total of 2,297 differentially expressed genes (DEGs) were identified; there were 1,023 upregulated and 1,274 downregulated genes in the MCD samples, with the |log_2_FC| values of the top ten upregulated genes (such as *NOD2*, *FOXM1*, and *CD53*) and downregulated DEGs (such as *ACTA1*, *PRTG*, and *KIT*) revealed in the volcano plot and heatmaps (Figures 2A,B).

Next, 797 ICA target genes were generated from the five databases (Supplementary Table S2). The ICA–ICA target gene network map showed the associations between ICA and the 797 predicted target genes. The pharmacological properties, toxicological reports, and 2D and 3D structures of ICA are presented in Table 2. The intersection of 2,297 DEGs, 3,278 MDRGs, and 797 ICA target genes was then used to obtain 45 differential MCD-ICA target genes for subsequent studies (Figure 2C).

**TABLE 2:** ICA pharmacological and toxicological reports.

Formula	C_33_H_40_O_15_	TPSA	238.20 Å^2^
MW	676.66 g/mol	Bioavailability score	0.17
Hato	48	Lipophilicity	0.69
a.Hato	16	BBB	−3
Rbon	9	MR	167.28
Hacc	15	Predicted LD_50_	5,000 mg/kg
Hdon	8		
Hepatotoxicity	Inactive	Mutagenicity	Inactive
Carcinogenicity	Inactive	Cytotoxicity	Inactive
Immunotoxicity	Active		
2D structure	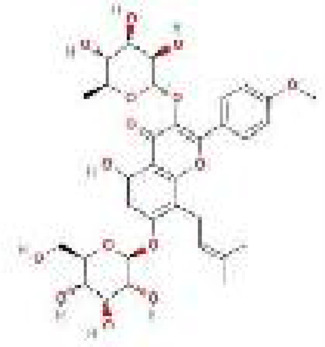	3D structure	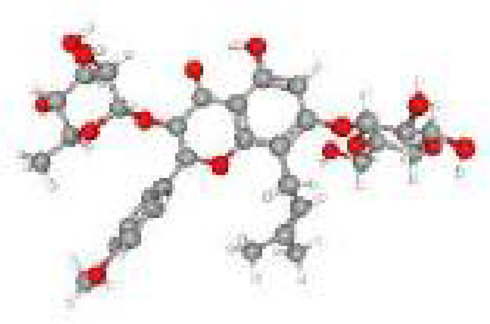

MW, molecular weight; Hato, number of heavy atoms; a.Hato, number of aromatic heavy atoms; Rbon, number of rotatable bonds; Hacc, number of hydrogen bond acceptors; Hdon, number of hydrogen bond donors; MR, molar refractivity; TPSA, topological polar surface area; BBB, blood–brain barrier.

In order to understand the function of the 45 differential MCD-ICA target genes, a Gene Ontology (GO) analysis was performed, which revealed that 19 candidate genes were associated with 552 GO signaling pathways, including 487 biological processes, 21 cellular components, and 44 molecular functions, demonstrating significant enrichment of five GO terms, such as the development of striated muscle tissue and the maturation of cardiac muscle tissue and the platelet alpha granule lumen (Figure 2D).

Kyoto Encyclopedia of Genes and Genomes (KEGG) enrichment analysis revealed 21 pathways, including the MAPK signaling pathway, the hematopoietic cell lineage, and the arginine biosynthesis (Figure 2E) (p < 0.05).

### 3.2 Identification of 19 candidate genes

**FIGURE 3:**
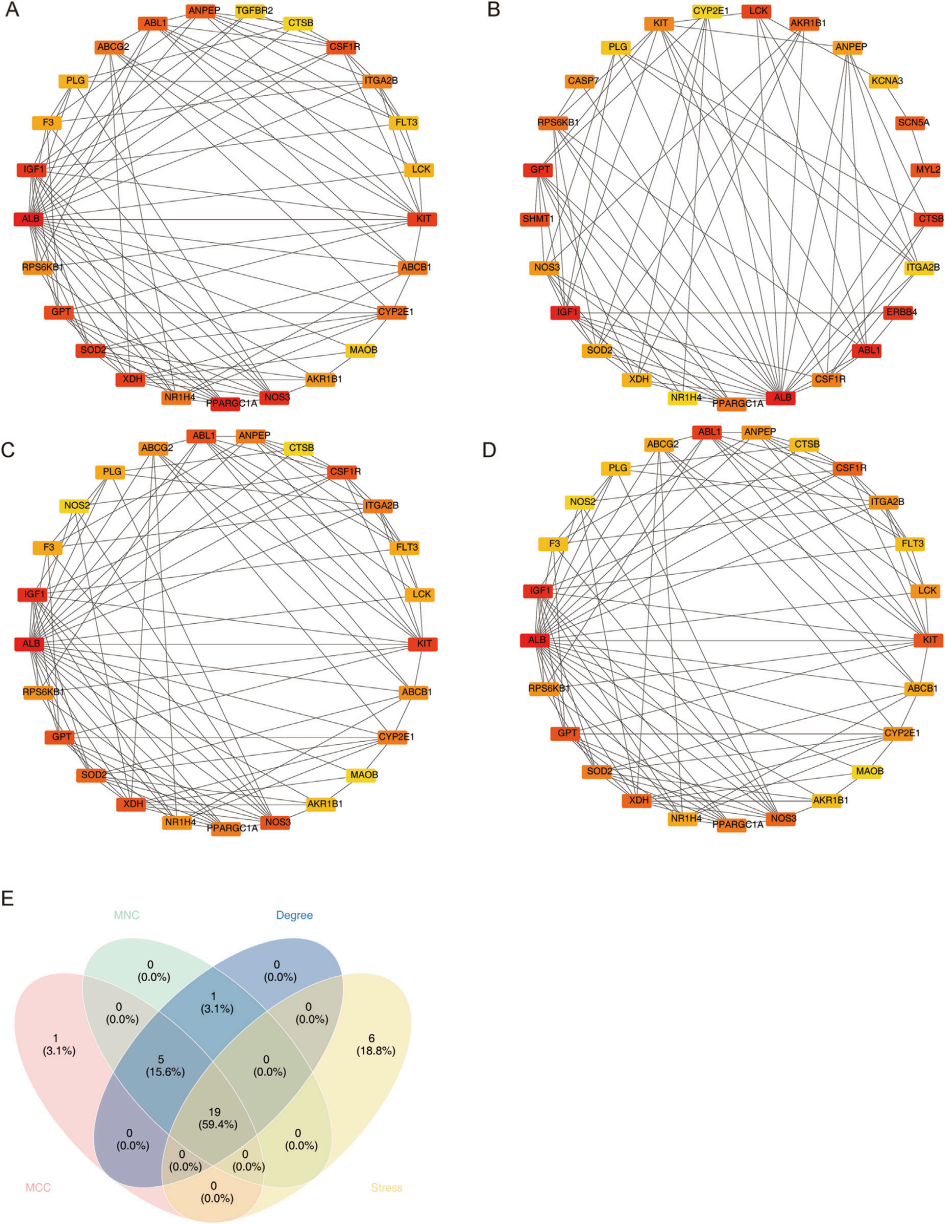
Four PPI network algorithms screen candidate genes. **(A)** MCC, **(B)** stress, **(C)** MNC, and **(D)** degree. **(E)** Intersection Venn graph of the four algorithms.

In order to obtain the candidate genes, 45 differential MCD-ICA target genes were used for protein–protein interaction (PPI) network construction (confidence = 0.4), and the genes with the top 25 scores in each algorithm were screened using four algorithms (MCC, stress, MNC, and degree); the results are shown in Figures 3A–D. The intersection of the genes screened using the four algorithms yielded a total of 19 candidate genes (Figure 3E).

### 3.3 Screening of key genes associated with MCD and validation

**FIGURE 4:**
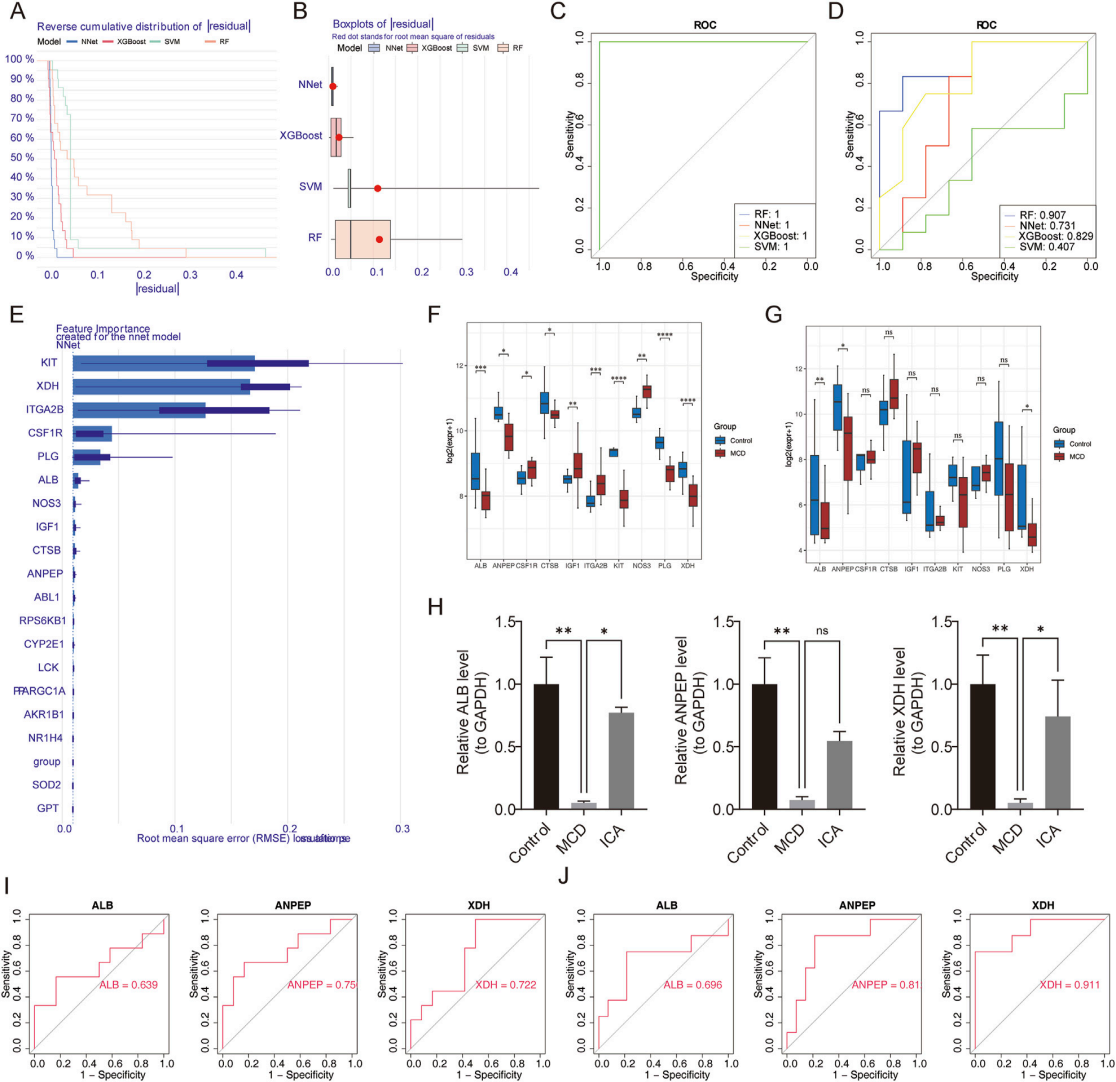
Screening of key genes. **(A,B)** Cumulative residual distribution plots and residual box plots of RF, NNet, XGBoost, and SVM models. **(C,D)** ROC curves of the four models in the training and validation sets. **(E)** Ranking of the importance of candidate genes in the NNet model. **(F,G)** Analysis of the expression levels of feature genes in the training and validation sets. **(H)** Expression of feature genes verified using RT-qPCR. **(I,J)** ROC curves of characteristic genes in the training and validation sets. ns represents p > 0.05, * represents p < 0.05, ** represents p < 0.01, *** represents p < 0.001, and **** represents p < 0.0001.

Using the 19 candidate genes, random forest (RF), neural network (NNet), extreme gradient boosting (XGBoost), and support vector machine (SVM) models were built with the training set. A comparison of the cumulative residual distribution plots (Figure 4A) and residual box line plots (Figure 4B) of the four models revealed that the NNet model corresponded to the smallest residual value.

Furthermore, the receiver operating characteristic (ROC) curves of the four models were plotted using the training (Figure 4C) and validation sets (Figure 4D). The area under the curve (AUC) value of the NNet model was greater than 0.7, indicating that the model had good predictive performance. Finally, the top ten genes in the NNet model were selected as feature genes (*KIT*, *XDH*, *ITGA2B*, *CSF1R*, *PLG*, *ALB*, *NOS3*, *IGF1*, *CTSB*, and *ANPEP*) (Figure 4E).

In order to obtain key genes of diagnostic significance in MCD, three genes (*ALB*, *ANPEP*, and *XDH*) with notable differences in expression and consistent trends between the MCD and control groups were obtained using the Wilcoxon test; these three genes were revealed to be downregulated in MCD (p < 0.05) (Figures 4F,G).

Similarly, reverse transcription quantitative polymerase chain reaction (RT-qPCR) analysis revealed that the expression of *ALB*, *ANPEP*, and *XDH* was obviously lower in the MCD samples than in the control samples and that the expression of *ALB* and *XDH* was significantly increased in the MCD samples after ICA intervention (p < 0.05) (Figure 4H).

Next, the ROC curves of the three genes were plotted, and the AUC values of the two genes in the training (GSE216841) and validation (GSE139061 and GSE246204) sets were found to be greater than 0.7. These two genes were defined as the key genes (*ANPEP* and *XDH*) and were used for subsequent analysis (Figures 4I,J).

### 3.4 Construction and evaluation of the nomogram

**FIGURE 5:**
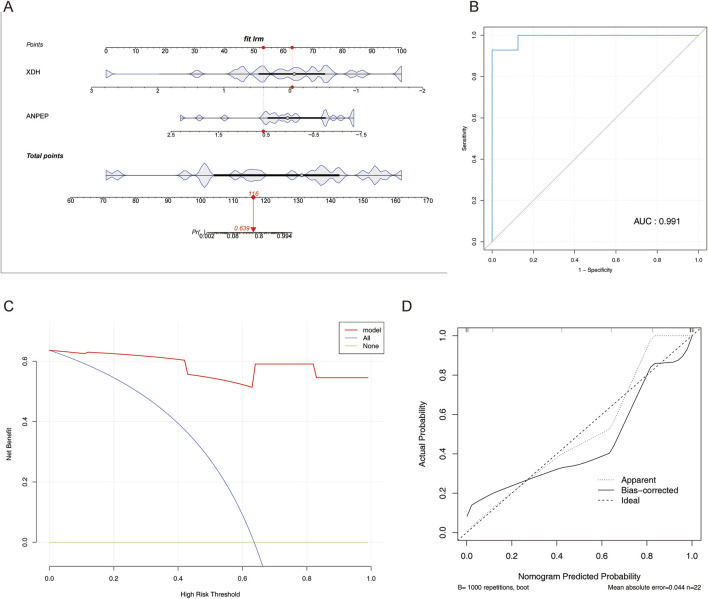
Construction and verification of the nomogram. **(A)** Nomogram constructed based on three key genes. **(B)** ROC curve of the nomogram. **(C)** DCA curve of the nomogram. **(D)** Calibration curve of the nomogram.

A nomogram allows the visualization of each predictor and its degree of influence on the outcome event. A nomogram model of the determined key genes was, therefore, constructed in this study to predict the probability of MCD (Figure 5A).

The AUC value was 0.991 (Figure 5B), and the DCA indicated that the nomogram model benefited from higher values than individual key genes (Figure 5C); slopes were close to 1 in the calibration curve (Figure 5D), all of which are indicative of the model’s good predictive effect.

### 3.5 Functional and correlation analyses of key genes

**FIGURE 6:**
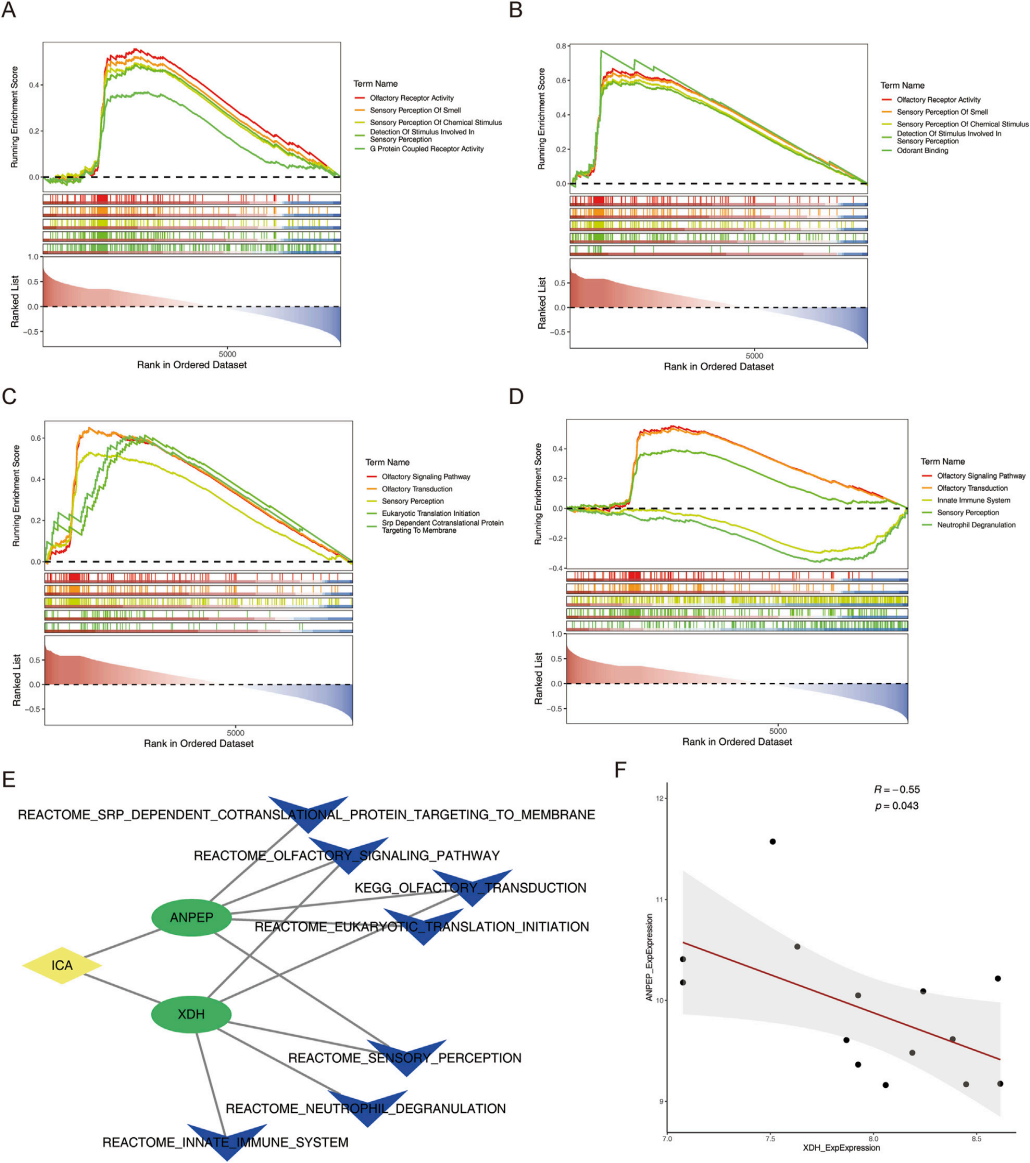
Functional and correlation analyses of key genes. **(A,B)** Single-gene GSEA of GO enrichment results of *ANPEP* and *XDH*. **(C,D)** Single-gene GSEA of KEGG enrichment results of *ANPEP* and *XDH*. **(E)** ICA–key gene–TOP5 KEGG pathway network map. **(F)** Correlation scatter plot of key genes in MCD samples.

In order to clarify the signaling pathways and biological functions associated with the key genes involved in MCD, gene set enrichment analysis (GSEA) revealed the top five pathways with significant enrichment of two key genes. In the single-gene GO enrichment analysis, the first three pathways of the *ANPEP* and *XDH* genes were olfactory receptor activity, sensory perception of smell, and sensory perception of chemical stimulus (Figures 6A,B) (p < 0.05).

In the single-gene KEGG enrichment analysis, the *ANPEP* gene was specifically enriched in the olfactory signaling pathway, olfactory transduction, and sensory perception. The *XDH* gene was specifically enriched in the olfactory signaling pathway, the olfactory transduction pathway, and the innate immune system pathway (Figures 6C,D) (p < 0.05).

In addition, to investigate the potential mechanisms through which ICA regulated the key genes, the relationships between key genes and KEGG pathways, which are based on the key genes, ICA, and the top five KEGG pathways of the key genes, the ICA–key gene–TOP5 KEGG (ICA–*ANPEP*–olfactory signaling pathway) pathway network map relationships (Figure 6E) were constructed. Next, the correlation between the key genes was assessed, the results of which revealed a substantial negative correlation between *ANPEP* and *XDH* in the training set MCD samples (cor = −0.55, p-value = 0.043) (Figure 6F).

### 3.6 Immune infiltration analysis

**FIGURE 7:**
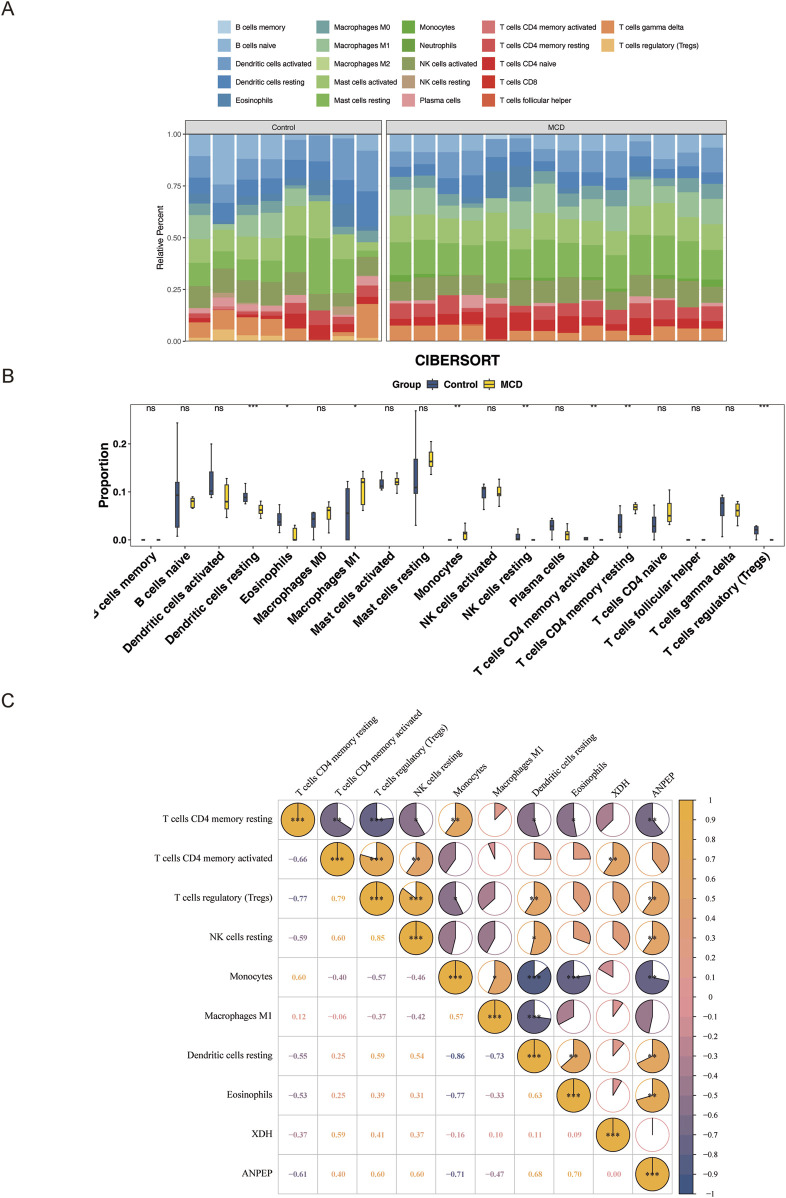
Immune infiltration analysis. **(A)** Relative proportion stacking chart of 22 types of immune cells in the training set. **(B)** Infiltration differences of 22 immune cells in MCD and control samples. **(C)** Correlation analysis between differential immune cells and key genes. From yellow to blue indicates the correlation from positive to negative; the darker the color, the stronger the correlation. ns represents p > 0.05, * represents p < 0.05, ** represents p < 0.01, and *** represents p < 0.001.

Immune cells are closely associated with the development of MCD (Ghamdi et al., 2020), and how immune cell infiltration occurs differently between the MCD and control groups was investigated. The infiltration scores of 22 immune cells in the samples from GSE216841 were determined using the CIBERSORT algorithm. The top three cells with the highest percentage of immune cells were resting natural killer (NK) cells (Figure 7A). In addition, eight immune cells, such as resting dendritic cells, eosinophils, and macrophages, were notably different (p < 0.05) between the MCD and control groups (Figure 7B).

Spearman analysis revealed positive correlations between regulatory T cells and resting NK cells (cor = 0.85, p-value = 0.001) and between eosinophils and *ANPEP* (cor = 0.70, p-value = 0.01), suggesting that these cells may be acting synergistically in certain biological processes. Furthermore, evidence confirmed that resting memory CD4 T cells and regulatory T cells (cor = −0.77, p-value = 0.001), along with monocytes and *ANPEP*, were the most negatively correlated (cor = −0.71, p-value = 0.01) (Figure 7C).

### 3.7 Association analysis of key genes with mitochondrial dysfunction and kidney tissue cells

**FIGURE 8:**
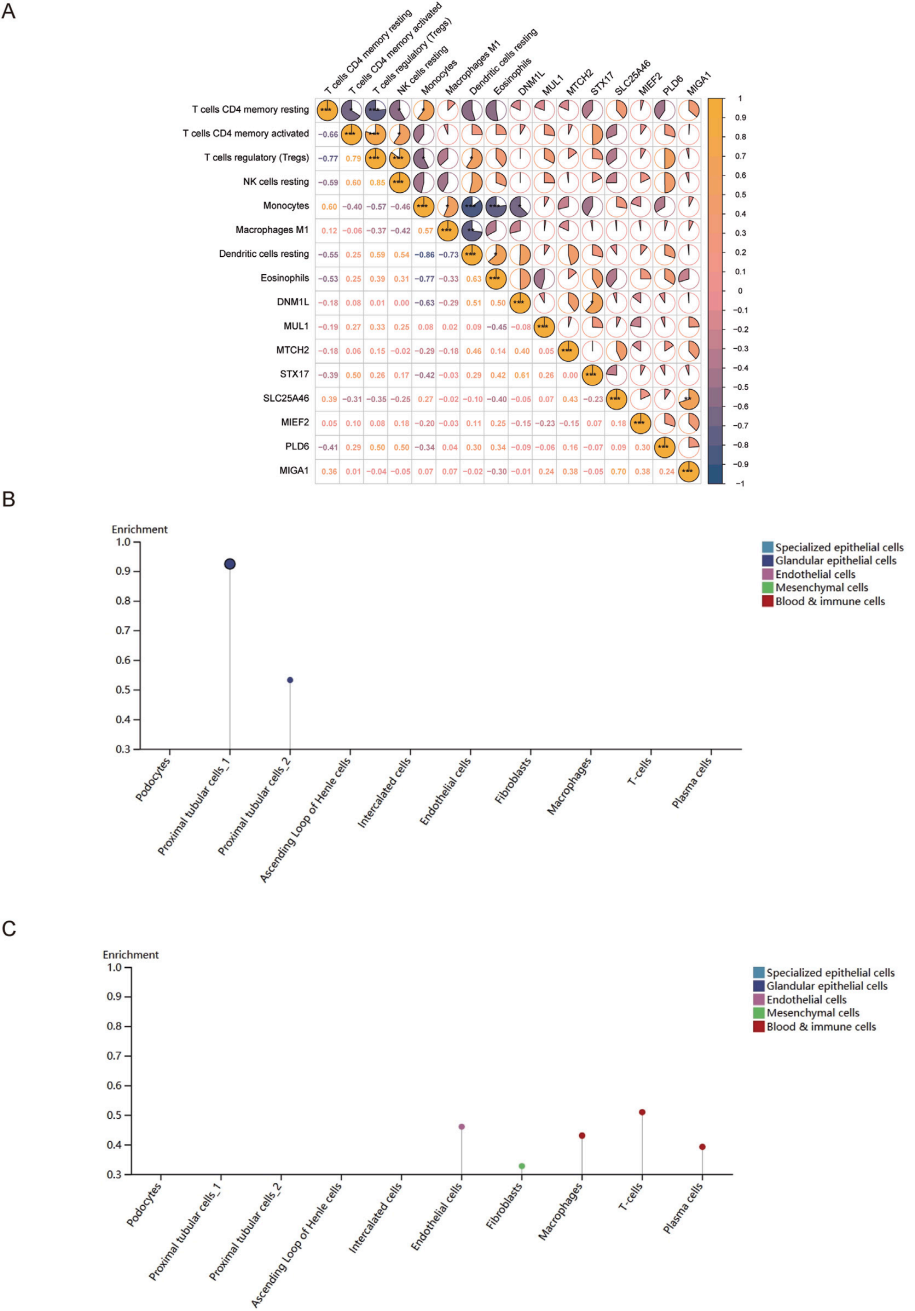
Association analysis of key genes with mitochondrial dysfunction and kidney tissue cells. **(A)** Correlation analysis between differential immune cells and mitochondrial dynamics genes. From yellow to blue indicates the correlation from positive to negative; the darker the color, the stronger the correlation. **(B,C)** Expression of *ANPEP* and *XDH* in different types of renal tissue cells.

Mitochondrial dynamics are regulated by fusion and fission proteins, both of which are important for organisms. Therefore, to explore the relationship between mitochondrial dynamics and the MCD immune microenvironment, the relationship between mitochondrial dynamics and the MCD immune microenvironment was explored in this study based on 23 genes related to mitochondrial dynamics. It was revealed that eight genes (*DNM1L*, *MIEF2*, *MUL1*, *SLC25A46*, *STX17*, *MIGA1*, *MTCH2*, and *PLD6*) were expressed in the training set samples, and DNM1L and differential immune cells (monocytes) were negatively correlated (cor = −0.63, p-value = 0.05), indicating an antagonistic role of DNM1L and monocytes in disease development (Figure 8A). Next, to determine the expression levels of key genes in nine kinds of renal tissue cells (podocytes, proximal tubular cells, *etc.*), the magnitudes of expression of key genes in renal tissue cells were analyzed. The results of the analysis revealed that *ANPEP* was expressed only in the proximal renal tubular cells (Figure 8B), and that *XDH* was expressed in five types of cells, including endothelial cells, fibroblasts, and macrophages (Figure 8C).

### 3.8 TF regulatory network and molecular docking

**FIGURE 9:**
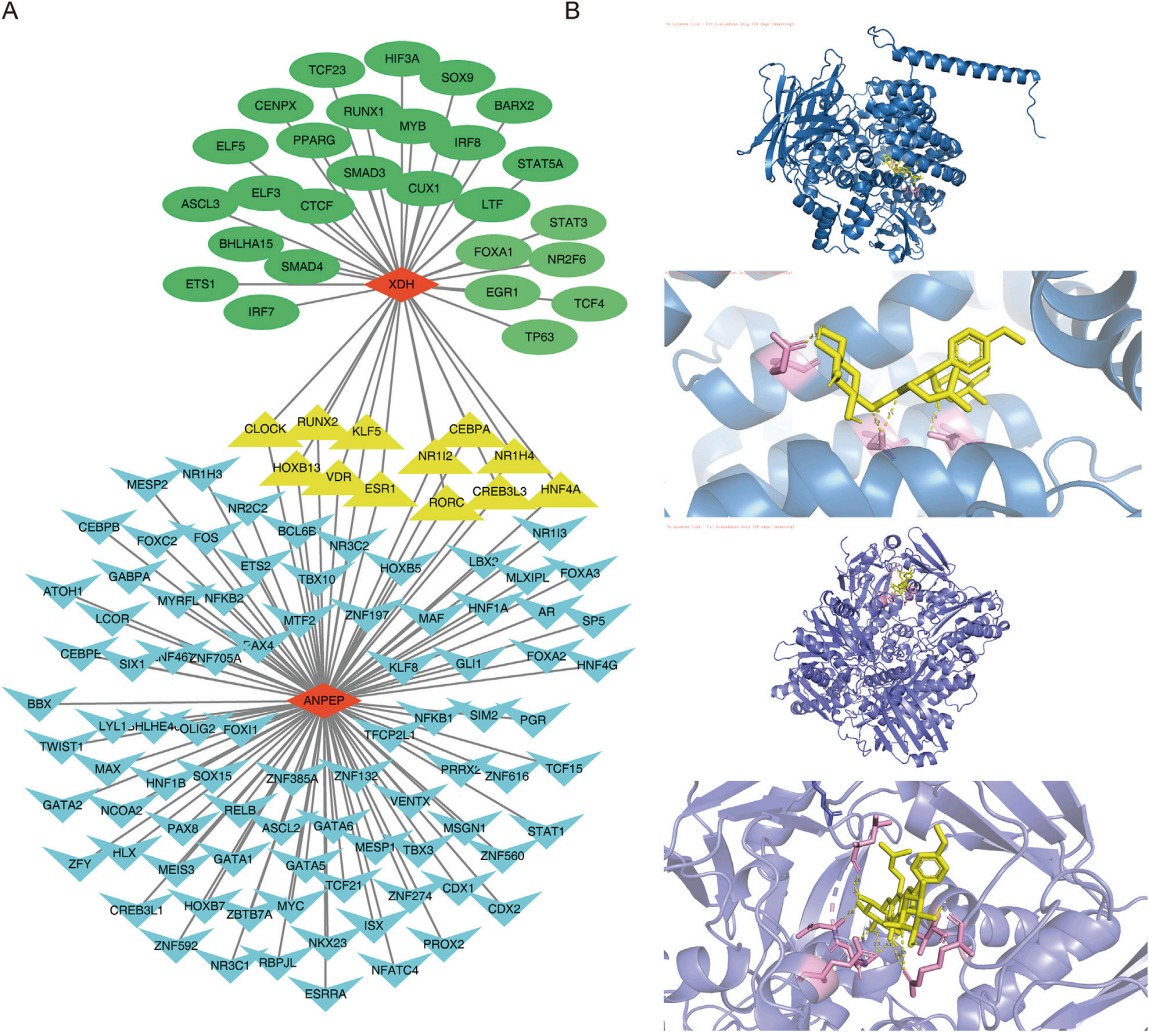
TF regulatory network and molecular docking. **(A)** TF–key gene regulatory network. Blue represents the TF of the *ANPEP* gene, and green represents the TF of the *XDH* gene. Yellow represents the TF shared by *ANPEP* and *XDH*, while red indicates the key gene. **(B)** Molecular docking of ICA and key genes.

Transcription factors specifically recognize the downstream target genes and build transcriptional complexes, thus playing important roles in regulating various biological processes. Therefore, to understand which TFs regulate key genes during disease development, TFs for the key genes were predicted. The results revealed that 98 TFs regulated *ANPEP* and 39 regulated *XDH*. Of these, 12 TFs co-regulated the genes *XDH* and *ANPEP*. A TF–key gene regulatory network was then constructed (KLF5–*XDH* and KLF5–*ANPEP*) (Figure 9A).

In order to further understand the role of ICA in relation to the key genes and determine the binding affinity of the compound ICA for key genes, the protein structures of the key genes *ANPEP* and *XDH,* were subjected to molecular docking with the molecular structure of ICA. The docking results revealed that the binding energy of ICA–*ANPEP* was −9.4 kcal/mol and that of ICA–*XDH* was −9.0 kcal/mol, both of which were below −5 kcal/mol. This indicates that the selected ICA has high binding affinity for the key genes *ANPEP* and *XDH* (Table 3; Figure 9B).

**TABLE 3:** Molecular docking results.

Key gene	Chemical compound	Binding energy
*ANPEP*	ICA	−9.4 kcal/mol
*XDH*	ICA	−9.0 kcal/mol

### 3.9 Effects of ICA on the renal tissue ATP levels in MCD rats

**FIGURE 10:**
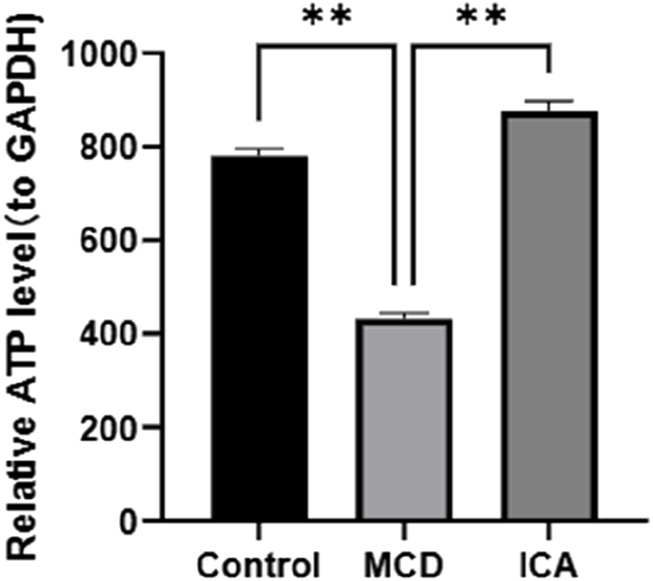
Expression of ATP in the colorimetric detections.

Colorimetric detection analysis revealed that ATP expression was obviously lower in the MCD samples than in the control samples and that ATP expression was significantly elevated in the MCD samples after ICA intervention (p < 0.01) (Figure 10).

### 3.10 Effects of ICA on renal mitochondrial ultrastructure in MCD rats

**FIGURE 11:**
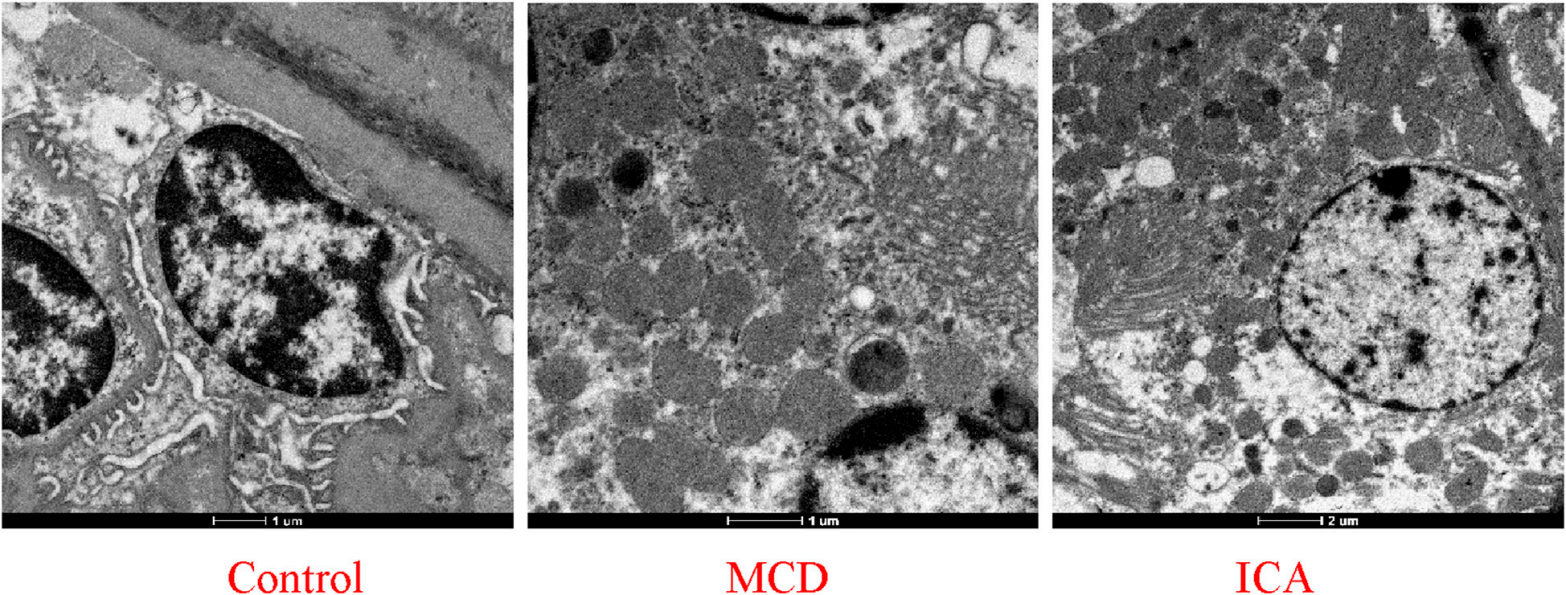
Mitochondrial structure in renal tissue under an electron microscope.

Transmission electron microscopy revealed that the ultrastructure of the mitochondria in the renal tissue from the control group was well preserved, with an intact outer membrane and cristae arranged in an orderly and continuous manner. In contrast, the mitochondria in the MCD group exhibited structural disorganization, including a shrunken and fragmented outer membrane and disordered or even absent cristae; some mitochondria also exhibited vacuolar degeneration. Compared with the MCD group, ICA resulted in a more intact outer membrane in the mitochondria (although some membranes remained slightly shrunken), restored crista integrity, and partially recovered the characteristic filamentous mitochondrial morphology (Figure 11).

## 4 Discussion

Minimal change disease (MCD) is a common pathological form of NS of unknown pathogenesis. Currently, the main treatments for MCD are glucocorticoids, immunosuppressants, and biological agents (Bensimhon et al., 2019; Gomez et al., 2024; Lan et al., 2024; Li et al., 2023). Mitochondria are highly important for eukaryotic aerobic respiration, and since renal oxygen consumption is high, the renal tissues of podocytes and tubular epithelial cells are rich in mitochondria (Wei and Szeto, 2019). Cellular function is energy-dependent and is sensitive to mitochondrial dysfunction, with the latter being one of the etiologies of glomerular and tubular diseases (Bhargava and Schnellmann, 2017; Gujarati et al., 2020). Studies have shown that ICA can inhibit inflammatory factors, maintain mitochondrial morphology, and thereby protect against renal function. This study used the transcriptome data of MCD and applied bioinformatics and network pharmacology, revealing two key genes (*ANPEP* and *XDH*) related to ICA therapy and mitochondrial dysfunction in MCD. In addition, the potential molecular mechanisms of these two key genes in MCD were explored. The findings provide a novel reference for the diagnosis and follow-up treatment of MCD patients.

ICA is an active monomeric compound extracted from the natural medicine *Epimedium* and exhibits multiple pharmacological effects, including regulating gut microbiota metabolism, alleviating ferroptosis, and activating autophagy (Liu et al., 2023; Wang et al., 2023; Bai et al., 2023). Research has indicated that ICA can alleviate cadmium-induced renal injury in rats by downregulating the TLR4/P2rx7/NF-κB signaling pathway, thereby suppressing the activation of the NLRP3 inflammasome, and this process may involve the synergistic action of multiple targets related to antioxidant and antiapoptotic effects (Zheng et al., 2024). The gut microbiota has been a major research focus in recent years. In prostate tumor models, the combined use of ICA and curcumol increased the quantity and richness of the gut microbiota and activated CD8^+^ T cells, thereby inhibiting the growth of cancer cells (Xu et al., 2024). Cao et al. (2024) reported that the natural medicine *Cornus officinalis* vinegar could alter the composition of the gut microbiota, regulating the size and number of lipid droplets in the liver tissue of high-fat diet-fed mice, and ultimately reduce steatosis. Overall, ICA may modulate the gut microbiota composition in the MCD, alter the host immune response, suppress inflammatory cytokine production, and attenuate renal inflammation. One randomized controlled trial revealed that the levels of ICA in humans are positively correlated with the levels of bone synthesis markers (such as bone-specific alkaline phosphatase (BSAP)), suggesting the therapeutic potential of ICA for treating osteoporosis (Yong et al., 2021). Therefore, it was speculated that various aspects of ICA are worthy of exploration in relation to the field of kidney disease.

*ANPEP* (alanyl aminopeptidase, membrane), also called “alanyl aminopeptidase,” is a membrane-associated extracellular enzyme located in the small intestine and kidney micromembranes and other plasma membranes. The gene encoding this enzyme has been shown to participate in several functions, including angiogenesis, tumor growth, and metastasis. The immunological response of this gene and any defects in it have been linked to various types of leukemia and lymphoma (Shui et al., 2019). *ANPEP* plays a role in glutathione (GSH) metabolism and exhibits broad substrate specificity. *ANPEP* is a part of the GSH metabolic pathway, in which it hydrolyzes the peptide L-cysteine glycine into cysteine and glycine substrates to resynthesize GSH(44). GSH is also an important factor in the synthesis of glutathione peroxidase 4 (GPX4). Evidence suggests that the blockage of GSH synthesis leads to a low expression of GPX4, thus reducing the antioxidant effect, and results in the accumulation of a large amount of ROS, which causes toxic reactions and initiates ferroptosis in cells (Su et al., 2019). Potential mechanisms underlying the relationship between type 2 diabetes and the *ANPEP* gene may involve the disruption of redox homeostasis and glutathione metabolism (Korvyakova et al., 2025). One study indicated that *ANPEP* downregulates basolateral *Na* ^*+*^ *-K* ^*+*^ *-ATPase* levels in proximal tubule cells through the *ANG IV/AGTRIV* signaling pathway. These findings suggest that *ANPEP* may contribute to renal ion dysregulation and the associated impairment of mitochondrial function (Kotlo et al., 2007). In addition, polymorphisms of the *ANPEP* gene are associated with diabetic microangiopathy (Korvyakova et al., 2024). The RT-qPCR results of this study revealed that the expression of *ANPEP* was markedly lower in the MCD group than in the control group and that the expression of *ANPEP* was greater in the MCD group than in the control group after ICA intervention.

*XDH* (xanthine dehydrogenase) is a set of molybdenum-containing hydroxylase enzymes involved in purine oxidative metabolism. The protein encoded by *XDH* has been identified as a moonlighting protein that can perform different functions (Bortolotti et al., 2021). Genetic deletion of the *XDH* gene in rats induces kidney damage, renal failure, and stunted growth and development. Transcriptomic analysis of the renal tissue has revealed several dysregulated pathways related to the lack of *XDH* expression, which are associated with the remodeling of inflammasomes, purinergic signaling, and redox homeostasis. Accumulating evidence suggests that *XDH* deficiency may affect kidney development through the dysregulation of epidermal growth factor (EGF) and its downstream STAT3 signaling (Dissanayake et al., 2024). Xdh-encoded xanthine oxidoreductase (XOR) plays a key role in purine metabolism by catalyzing the oxidation of hypoxanthine to xanthine, which in turn oxidizes xanthine to uric acid (Bortolotti et al., 2021; Furuhashi, 2020). This process will produce reactive oxygen species (ROS) such as superoxide anion; excessive ROS production greater than the removal capacity of the cell can cause oxidative stress and mitochondrial damage, resulting in mitochondrial dysfunction (Thies et al., 2023). For XDH to participate in the purine metabolism, a purine metabolic disorder may affect the cell energy metabolism and redox state, which affects the function of the podocyte (Thies et al., 2023). Xanthine metabolism mediated by *XDH* may be linked to oxidative stress, ultimately contributing to mitochondrial dysfunction. In this study, RT-qPCR analysis revealed that the expression of *XDH* in the MCD samples was obviously lower than that in the control samples and that the expression of *XDH* was significantly greater after ICA intervention than in the MCD samples. The diagnostic value of *XDH* in MCD was thus verified.

GSEA results revealed that the key genes identified were enriched in the olfactory signaling pathway, which is responsible for detecting inhaled odor molecules. This finding suggests a role for the olfactory signaling pathway in MCD. Olfactory receptors (ORs), primarily known as odor sensors in the olfactory epithelium within the olfactory signaling pathway, are also expressed in non-sensory tissues such as the kidney, where they contribute to normal renal physiology (Kalbe et al., 2016; Motahharynia et al., 2022). For instance, renal ORs are implicated in blood pressure regulation and the response to acidemia (Shepard, 2021). Abnormal OR activation may impair glomerular podocyte function, compromise the filtration barrier stability, and potentially induce MCD. However, the link between the olfactory signaling pathway and MCD remains preliminary and speculative; future functional studies are required to elucidate its exact role.

The transcription factor KLF5 can co-regulate *ANPEP* and *XDH*. KLF5 is a member of the Kruppel family of factors that regulate many cellular functions, such as apoptosis, proliferation, and differentiation (Luo and Chen, 2021). Moreover, KLF5 can regulate renal cell proliferation, podocyte apoptosis, renal fibrosis, renal tubulointerstitial inflammation, and other diseases (Liu et al., 2024; Xu et al., 2020). Overexpression of KLF5 in podocytes prevents PAN-induced cell cycle arrest and podocyte apoptosis by blocking the activation of the ERK/p38 MAPK pathway (Li et al., 2018; Li et al., 2021).

In this study, two key genes (*ANPEP* and *XDH*) associated with ICA therapy and MDRGs were identified in MCD, and the RT-qPCR results confirmed these results. In addition, the biological pathways associated with the key genes were identified, and the potential molecular mechanisms and the expression of the key genes in kidney tissue cells were explored using single-gene GSEA, immune infiltration analysis, clinical modeling, molecular docking, transmission electron microscopy, and colorimetric detection. These findings provided a novel reference for the diagnosis and follow-up treatment of MCD patients. However, this study focused primarily on the use of bioinformatics analysis and network pharmacology analysis. The generalizability of the results may also be limited by the small sample size used in the study. Additionally, comprehensive pathway validation experiments to confirm the roles of the identified key genes are lacking. The investigation and further validation of the mechanism by which ICA interferes with mitochondrial dysfunction in MCD will be continued through additional *in vivo* and *in vitro* experiments. Additionally, we plan to conduct further *in vivo* and *in vitro* experiments to validate icariin’s therapeutic role in MCD.

## Data Availability

The datasets analyzed for this study can be found in the GEO database, GeneCards database, PubChem database, Comparative Toxicogenomics Database, UniProtKB database: https://www.ncbi.nlm.nih.gov/geo/, https://www.genecards.org/, https://pubchem.ncbi.nlm.nih.gov/, https://ctdbase.org/, https://www.UniProt.org/.
